# Transcriptome analysis to explore the mechanism of downregulated TNIK influencing the effect of risperidone

**DOI:** 10.3389/fphar.2024.1431923

**Published:** 2024-08-23

**Authors:** Ruixue Yuan, Yaojing Li, Xiangyi Li, Yingmei Fu, Ailing Ning, Dongxiang Wang, Ran Zhang, Shunying Yu, Qingqing Xu

**Affiliations:** ^1^ Shanghai Key Laboratory of Psychotic Disorders, Shanghai Mental Health Center, Shanghai Jiao Tong University School of Medicine, Shanghai, China; ^2^ 958 Hospital of PLA ARMY, Chongqing, China; ^3^ Bio-X Institutes, Key Laboratory for the Genetics of Developmental and Neuropsychiatric Disorders (Ministry of Education), Shanghai Jiao Tong University, Shanghai, China

**Keywords:** risperidone, Traf2 and Nck-interacting protein kinase (TNIK), transcriptome analysis, protein-protein interaction, bone mineralization

## Abstract

**Background:**

Risperidone is one of the most reliable and effective antipsychotics for schizophrenia treatment. However, the mechanism of action of risperidone is not yet fully understood. Traf2 and Nck-interacting protein kinase (*TNIK*), a schizophrenia susceptibility gene, is associated with risperidone treatment response. Our previous *in vitro* experiments confirmed that downregulated TNIK affected the effect of risperidone on downstream targets. However, the effect of downregulated TNIK on risperidone-induced molecular expression remains to be further explored.

**Methods:**

Transcriptome analysis was performed on U251 cells subjected to risperidone, *TNIK* siRNA, and no treatment, respectively. Compared to the no-treatment group, two groups of DEGs were screened out and then intersected with the schizophrenia-related genes to screen the cross-talk genes. Those DEGs were analyzed using GO and KEGG. STRING and Cytoscape were used to construct a protein-protein interaction (PPI) network for the cross-talk gene.

**Results:**

The results showed that the parathyroid hormone synthesis, secretion, and action were significantly enriched after risperidone treatment. Downregulated TNIK could have an impact on the collagen-containing extracellular matrix, signaling receptor activator activity, and PI3K-Akt signaling pathway. Interestingly, bone mineralization function and calcium signaling pathway were enriched in the cross-talk genes. Additionally, FGFR2, FGF1, and FGFR might be the potential targets for *TNIK* affecting the effects of risperidone.

**Conclusion:**

The study indicated that risperidone primarily influences functions and/or pathways associated with bone metabolism, potentially contributing to the adverse effect of osteoporosis. Our study may offer a novel perspective on investigating the mechanisms underlying the adverse effects of risperidone.

## 1 Introduction

Schizophrenia (SCZ) is a chronic psychiatric illness characterized by psychotic symptoms including hallucinations, cognitive impairment, and delusions, leading to a social and occupational decline in many case (Hung et al., 2021; Jauhar et al., 2022). Patients usually need long-term medication to relieve the symptoms. Currently, antipsychotic drugs are the primary treatment for patients with SCZ via blocking dopaminergic and serotonergic receptors, but there is high inter-individual variability in terms of therapeutic benefits and side effects (Heide et al., 2016; Morozova et al., 2022). Approximately 20%–30% of SCZ patients are resistant to antipsychotic treatment and less than 40% achieve remission (Lisoway et al., 2021).

Risperidone (RPD), one of the widely prescribed antipsychotics for schizophrenia treatment, is a selective monoaminergic antagonist drug. It can be used to treat both positive and negative symptoms of SCZ, as well as alleviate the emotional symptoms associated with SCZ (Arani et al., 2023). However, there are a variety of side effects associated with RPD treatment, including weight gain, extrapyramidal symptoms, prolactin elevation, and osteoporosis (Zheng et al., 2019; Arani et al., 2023). For osteoporosis, many studies have suggested that long-term treatment with RPD increases the risk of osteoporosis by elevating serum prolactin levels (De Hert et al., 2016; Zheng et al., 2019; Chen Y. et al., 2021). Specifically, a recent study about 250 inpatients with schizophrenia receiving risperidone monotherapy has shown that patients have a high rate of osteoporosis (Chen Y. et al., 2021). However, Clapham et al. argued that RPD does not appear to be linked to an increased risk of osteoporosis-related fractures based on a population-based study (Clapham et al., 2020). Therefore, there is inconsistency among the available studies regarding the causal relationship between RPD and osteoporosis. The complex of therapeutic and adverse effects of RPD may derive from the variety of receptor targets. RPD treatment has multiple receptor actions, including blockade of serotonin 2A, 2C and 7 receptors and dopamine D2 receptor, α1 and α2-adrenergic receptors antagonism, and inverse agonist actions at histamine H1 receptors (Amodeo et al., 2014; Zheng et al., 2019). The blocking effect on dopamine D2 receptor can elevate prolactin secretion (Peuskens et al., 2014), which may be linked to elevated risk of osteoporosis. However, the effects of RPD are also influenced by other genetic factors. For example, Heide J et al. have reported that genotypes associated with increased Kv11.1-3.1 expression play a positive role in the therapeutic action of RPD (Heide et al., 2016). Two single-nucleotide polymorphisms (SNPs) rs155333 and rs6465938 of *RELN* were found to be associated with RPD response in the Chinese Han population (Xu et al., 2020). The study also revealed that apart from the previously identified genes *CYP2D6*, *ABCB1*, *DRD3*, and *HTR2C*, several new candidate genes, including *TNIK*, *RELN*, *NOTCH4*, and *SLC6A2*, were also identified to be associated with antipsychotic treatment response, including for RPD (Xu et al., 2016).

Since it is well established that genetic factors can influence the efficacy of RPD and may also partially explain the variation of treatment between patients (Yu et al., 2018), a better understanding of the genetic risk factors involved in medication response may offer potential guidance for clinicians in choosing a personalized and optimized medication based on their genetic profiles. Traf2 and Nck-interacting protein kinase (*TNIK*) may be a promising target to influence the efficacy of antipsychotics, including RPD. A genome-wide association study (GWAS) about Chinese Han inpatients with SCZ revealed five novel loci has shown that significant genome-wide association with general antipsychotic treatment response, one of which was rs6444970 in *TNIK* (*p* = 4.85 × 10^−8^) (Yu et al., 2018). Based on our preliminary study, we identified an association between several genes, including *TNIK*, and four antipsychotic drugs: risperidone, clozapine, quetiapine, and chlorpromazine (Xu et al., 2016). Subsequently, we specifically confirmed that variants rs2088885 and rs7627954 in *TNIK* were significantly associated with risperidone treatment response in a cohort of 148 unrelated inpatients who received risperidone monotherapy (Xu et al., 2022). Moreover, our *in vitro* cell experiments suggested that downregulated TNIK indeed affected the effects of RPD on downstream targets (Yuan et al., 2021). However, there is limited research investigating the mechanism by which the downregulation of TNIK impacts the effect of RPD.

*TNIK* encodes a serine/threonine kinase of the germinal center kinase (GCK) family and contains a scaffold domain that is important in the regulation of cell proliferation and postsynaptic signaling (Coba et al., 2012; Nie et al., 2021). It is found to be highly expressed in the human nervous system, and *in vivo* animal study has shown that *Tnik* plays a critical role in synapse and nucleus function within the nervous system and is a crucial regulator for cognitive function (Coba et al., 2012). In patients with SCZ, *TNIK* mRNA has been confirmed to be upregulated in the dorsolateral prefrontal cortex (Potkin et al., 2010). TNIK is also found as a crucial synaptic partner for the known psychiatric risk-associated protein DISC1 to regulate synapse composition and activity via DISC1–TNIK interaction (Wang et al., 2011). In addition, the methylome-wide association study of first-onset SCZ patients in Chinese Han population integrated with GWAS has identified *TNIK* as a susceptibility gene of SCZ (Nie et al., 2021). Hence, these reports support a role for *TNIK* as a risk factor for SCZ, as well as a potential target for RPD.

Overall, RPD, an atypical antipsychotic agent, has limited research on drug metabolic pathways and genetic risk factors influencing drug effect. *TNIK*, as a susceptibility gene of SCZ, has been reported to associate with RPD treatment response, but the potential mechanisms underlying downregulated *TNIK* affects RPD’s effect have been not or rarely investigated. Therefore, the purposes of this study were to discover the transcriptome profile of RPD and TNIK knockdown respectively, and then further explore the potential mechanism of TNIK influencing the molecular expression of RPD. To address this issue, we intervened pharmacologically with the appropriate concentration of RPD and downregulated the expression of *TNIK* by siRNA in human glioma U251 cells, respectively. Finally, we extracted the RNA of different groups for ribonucleic acid sequencing (RNA-seq) to identify the differentially expressed genes (DEGs) and gain more insight into the potential mechanisms.

## 2 Materials and methods

### 2.1 Cell culture and treatment

U251 (human glioma) cell line was purchased from the Cell Bank of Shanghai Institute of the Chinese Academy of Sciences and cultured in a humidified 5% CO_2_ incubator at 37°C in Dulbecco’s Modified Eagle’s Medium (DMEM, Thermo Scientific) supplemented with 10% fetal bovine serum (FBS, Thermo Scientific) and 1% penicillin/streptomycin (GIBCO). For investigating the effect of downregulated TNIK, the cells were cultured in 6-well plates and transiently transfected with hTNIK small interfering RNAs (siRNAs, Shanghai dnabio company) (knockdown group) using Lipofectamine 3,000 kit (Invitrogen) at 70% confluency. To identify the role of RPD, we determined the half inhibitory concentration (IC50) of RPD using MTT cell proliferation assay kit (Thermo Scientific) as described previously (Yuan et al., 2021), and the result showed the IC50 of RPD was 111.3 μM. Then, RPD treatment with 111.3 μM was performed on U251 cells (RPD group) for 24 h. The control group was constructed without treatment. All assays were performed in triplicate in at least three independent experiments.

### 2.2 Detection of mRNA level by qRT-PCR

The *TNIK* siRNA sequences were: 5′-GCA​AGA​AAG​AGA​CUA​CUU​ATT-3′ (sense) and 5′-UAA​GUA​GUC​UCU​UUC​UUG​CTT-3′ (anti-sense). To assess the knockdown efficiency of *TNIK* siRNA, we quantified the expression level of *TNIK* mRNA using qRT-PCR, with *GAPDH* serving as the endogenous reference gene. The primers used were as follows: *TNIK*, forward 5′- GGT​AGA​AGA​ACG​GTC​AAG​GCT​CAA​C-3′ and reverse 5′- GGC​TGA​ACT​CCA​CTA​ATG​CTG​AAG​G-3′; *GAPDH*, forward 5′- CAG​CCT​CAA​GAT​CAT​CAG​CAA​TG-3′ and reverse 5′- CAT​GAG​TCC​TTC​CAC​GAT​ACC-3′. Total RNA was extracted using RNA extraction kit (DP419, TIANGEN). Next, RNA was reverse transcribed into cDNA with PrimeScript™ RT reagent Kit with gDNA Eraser (TaKaRa) according to the instructions. SYBR^®^ Green Realtime PCR Master Mix (TOYOBO) was applied for PCR amplification with Roche LightCycler 480. Reactions were carried out with the following amplification conditions: 95°C for 30 s, followed by 40 cycles of reaction at 95°C for 5 s, 55°C for 10 s, and 72°C for 15 s. Last, a melting curve was performed after the amplification cycle to confirm the specificity of the amplification reaction. The relative mRNA expression levels of genes were calculated using the 2^−ΔΔCT^ method. PCR reactions were performed in triplicate.

### 2.3 RNA preparation

After transfection or drug treatment, total cell RNA from the three groups (control, knockdown, and RPD) was extracted respectively using RNA extraction kit (DP419, TIANGEN) according to the manufacturer’s instructions. 1% agarose gels were used to monitor the degradation and contamination of RNA samples. The purity of RNA was examined by NanoPhotometer^®^ spectrophotometer (IMPLEN, CA, United States) and the integrity was accurately determined using the RNA Nano 6000 Assay Kit of the Bioanalyzer 2100 system (Agilent Technologies, CA, United States).

### 2.4 Library construction

The RNA-seq library was constructed in line with the manufacturer’s protocol of NEBNext^®^ UltraTM RNA Library Prep Kit for Illumina^®^ (NEB, United States) and index codes were added to attribute sequences to each sample. After the purification of total RNA with poly-T oligo-attached magnetic beads, mRNA was obtained and subsequently fragmented into small pieces using divalent cations under elevated temperature in NEBNext First Strand Synthesis Reaction Buffer (×5). First-strand complementary DNA (cDNA) was transcribed from the fragmented mRNA using random hexamer primer and M-MuLV Reverse Transcriptase. Second-strand cDNA was synthesized with dNTPs in DNA polymerase I system after the degradation of the RNA strand with RNase H. Purified double-stranded cDNA then went through end reparation and 3′ ends adenylation, followed by ligation of NEBNext adaptor with hairpin loop structure. DNA fragments around 250–300 bp were screened for PCR amplification with AMPure XP beads. PCR products were purified using AMPure XP beads to create the final complementary DNA library, and library quality was assessed on the Agilent Bioanalyzer 2100 system. Lastly, after clustering the index-coded samples on a cBot Cluster Generation System using TruSeq PE Cluster Kit v3-cBot-HS (Illumia), paired-end sequencing of the sequencing library was performed in Illumina Novaseq platform and 150 bp paired-end reads were generated.

### 2.5 Identification and analysis of DEGs

Before data analysis, raw reads should be pre-processed to remove reads containing adapter, reads containing poly-N, and low-quality to obtain clean reads with high quality for subsequent analyses. Reference genome and gene model annotation files were downloaded from the genome website, and Hisat2 v2.0.5 was used to build the index of the reference genome and align paired-end clean reads to the reference genome. The expression level of genes in each sample was estimated using Fragments Per Kilobase Per Million Mapped Fragments (FPKM) values. Differential expression analysis was performed in edgeR package, which is based on the statistical test of the dispersion of negative binomial distributions of genes by fitting a generalized linear model with likelihood ratios to identify DEGs. Bonferroni correction was used for multiple tests and p.adj < 0.05 was considered significant. Furthermore, we also calculated the fold change by comparing two different groups, and |log2 (fold change)| > 1 was set as an additional condition to screen for DEGs. In general, the screening criterion for DEGs was to satisfy both p.adj < 0.05 and |log2 (fold change)| > 1. The ggplot2 package was used to draw the volcano plot to display the distribution of DEGs.

### 2.6 Functional enrichment analysis

To better investigate the underlying biological mechanism and pathways, all DEGs were subjected to Gene Ontology (GO) and Kyoto Encyclopedia of Genes and Genomes (KEGG) enrichment analyses via clusterProfiler packages in R platform. For GO analysis, it was classified into three subgroups, including biological process (BP), cellular component (CC), and molecular function (MF). p.adj < 0.05 was set as the threshold.

### 2.7 Construction of the protein-protein interaction (PPI) network

Firstly, we obtained the SCZ-related gene from the following sites: GeneCards (https://www.genecards.org/), OMIM (https://www.omim.org/) and DisGeNet (https://www.disgenet.org/). We subsequently made the Venn diagram in R platform by intersecting the SCZ-associated genes with the DEGs of RPD vs. control (R vs. C) and knockdown vs. control (K vs. C). The cross-talk genes of these three groups were then used to perform the GO and KEGG enrichment analysis. Subsequently, the STRING database (https://cn.string-db.org/) was used to construct the PPI network of cross-talk genes. After that, the PPI network was revisualized using Cytoscape 3.10.1 software. Firstly, we hid the unconnected genes and then calculated the degree score with the network analysis function. Finally, the PPI network was re-adjusted based on the degree score. The larger the circle and the darker the color, the higher the degree score.

## 3 Results

### 3.1 Detection of mRNA expression

As the qRT-PCR result indicated, transfection of *TNIK* siRNA in U251 cells significantly reduced the mRNA levels of *TNIK* (Supplementary Figure S1).

### 3.2 Identification of the DEGs

**FIGURE 1:**
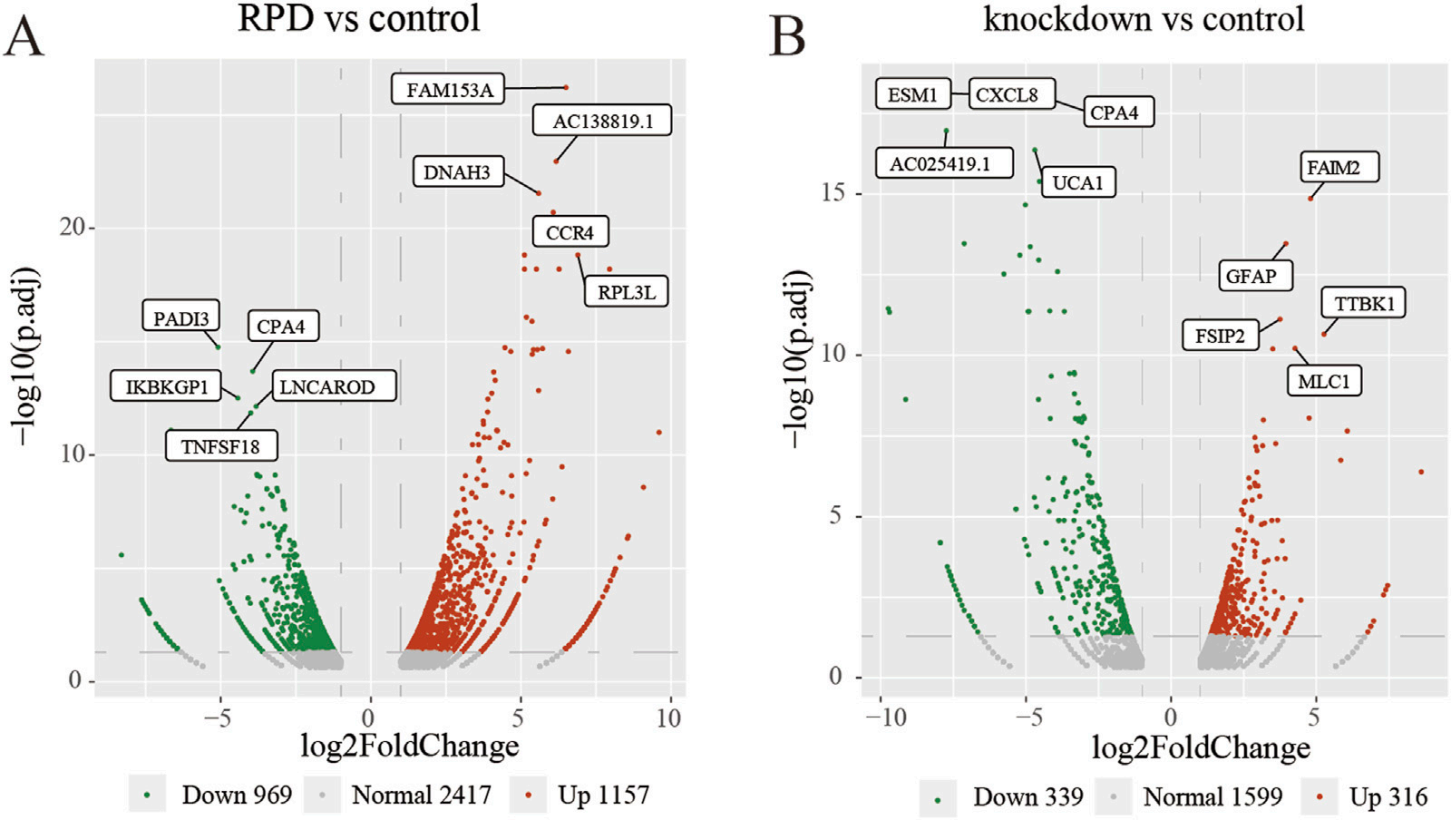
Volcano plots of DEGs. The top 5 upregulated and downregulated genes with the smallest *p*-adj value were marked on the plot. **(A)** Volcano plot of all DEGs between RPD group and control group. **(B)** Volcano plot of all DEGs between *TNIK* knockdown group and control group. p.adj < 0.05 was considered significant.

This study aimed to investigate the molecular mechanism of RPD and downregulated TNIK. Additionally, we also explored the potential functions and/or pathways involved in the downregulation of TNIK that may impact the effects of RPD. High-throughput sequencing was conducted, followed by a DEG analysis to identify changes in gene expression. Compared with the control group, the volcano plot showed that a total 2,126 DEGs with p.adj < 0.05 and |log2 (fold change)| > 1 were obtained in RPD group, of which 1,157 were upregulated while 969 downregulated (Figure 1A). In the meanwhile, data analysis of control and knockdown groups obtained 316 significantly upregulated genes and 339 significantly downregulated genes (Figure 1B). The results indicated that RPD treatment induced greater changes in gene expression than *TNIK* knockdown in U251 cells.

### 3.3 Functional analysis of DEGs

**FIGURE 2:**
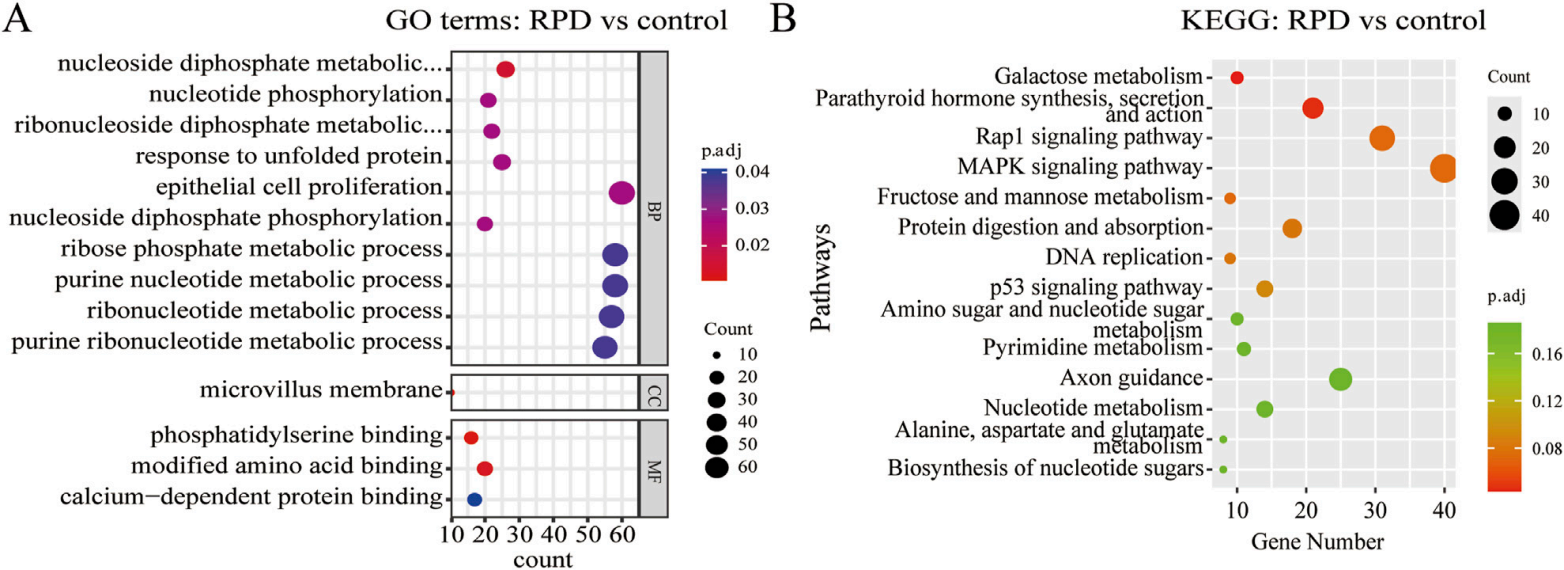
GO and KEGG enrichment analysis of DEGs between RPD group and control group. **(A)** The bubble plot of GO analysis showed the top 10 processes in BP, the only 1 process in CC, and the only three processes in MF. **(B)** The bubble plot of KEGG analysis showed only the first two pathways were significantly enriched by DEGs. p.adj < 0.05 was considered significant.

**FIGURE 3:**
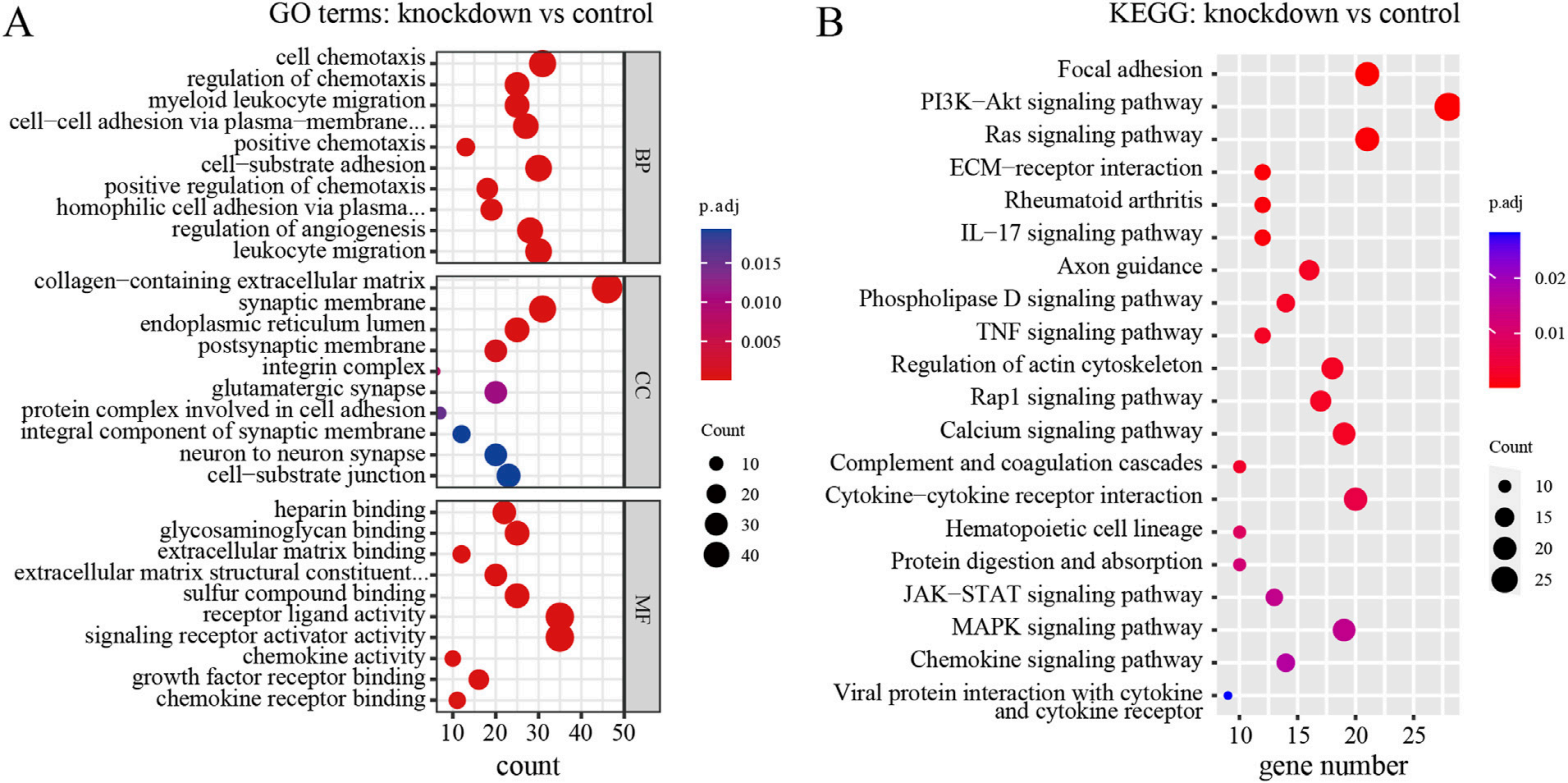
GO and KEGG enrichment analysis of DEGs between *TNIK* knockdown group and control group. **(A)** The result of GO analysis showed the top 10 processes enriched by DEGs in BPs, CCs, and MFs. **(B)** The result of KEGG analysis showed the top 20 pathways enriched by DEGs. p.adj < 0.05 was considered significant.

To explore the biological functions and pathways of these DEGs, we performed GO and KEGG enrichment analyses. Firstly, we observed the comparison between RPD and control groups. According to the GO enrichment result, 32 BP terms, 1 CC term, and 3 MF terms were significantly enriched in DEGs. The most significantly enriched terms include nucleoside diphosphate metabolic process and modified amino acid binding (Figure 2A). In addition, a total of 697 genes were mapped to the signaling pathways. Only two KEGG pathways were significantly enriched, including galactose metabolism and parathyroid hormone synthesis, secretion, and action (Figure 2B). Then, for the comparison of knockdown and control groups, 289 BP terms, 19 CC terms, and 26 MF terms were identified to be significantly enriched in GO analysis, and the top 10 processes among these three term were displayed in Figure 3A. Among the BP terms, most genes were enriched in cell chemotaxis, cell-substrate adhesion, and leukocyte migration. Among the CC terms, the most enriched terms were mainly related to collagen-containing extracellular matrix, synaptic membrane, and endoplasmic reticulum lumen. Among the MF terms, most genes were categorized as receptor-ligand activity, signaling receptor activator activity, glycosaminoglycan binding, and sulfur compound binding. Further to this, 21 KEGG pathways were enriched in total, mainly including focal adhesion, PI3K-Akt signaling pathway, and Ras signaling pathway (Figure 3B).

### 3.4 Identification of the hub genes

**FIGURE 4:**
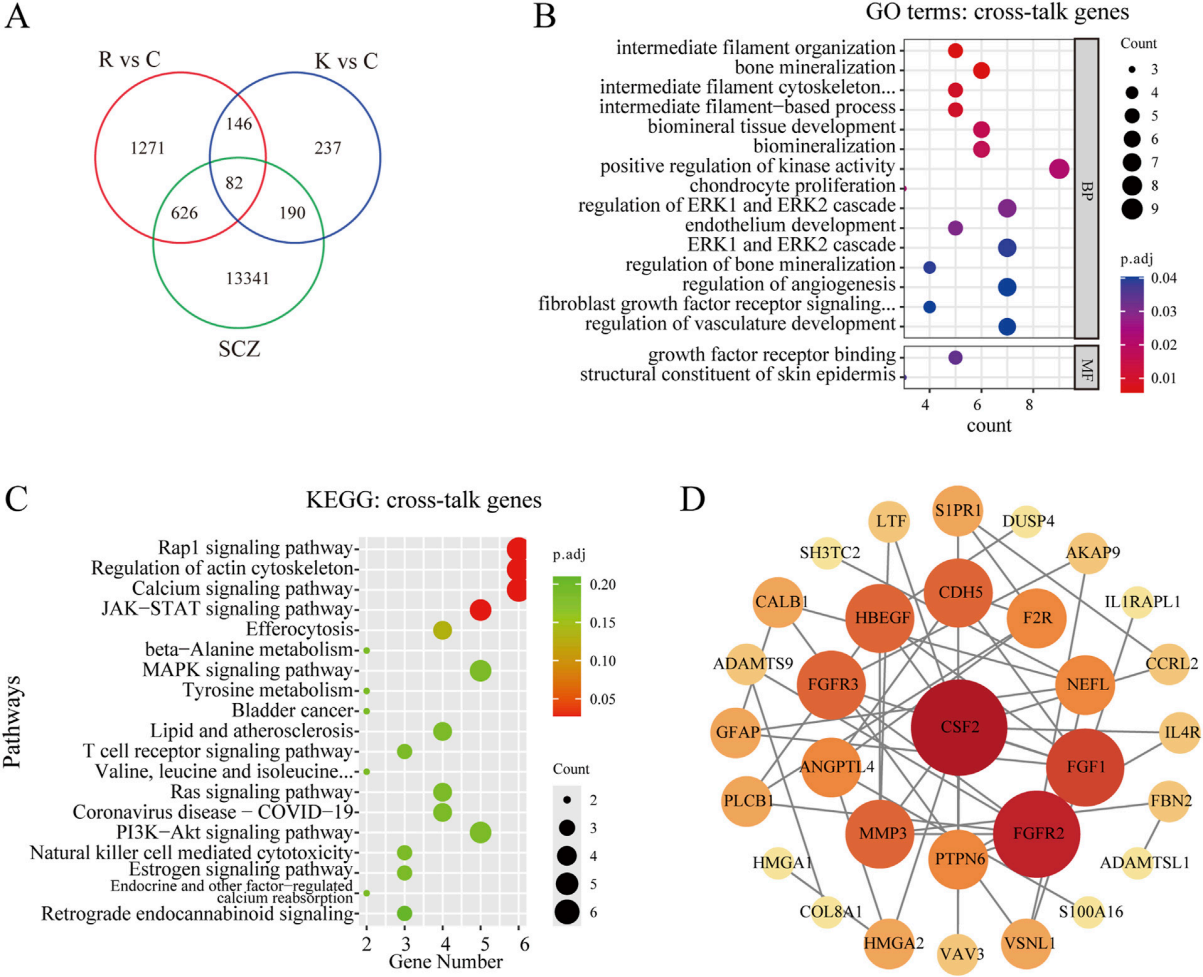
Screening and analyzing the cross-talk genes, and constructing the PPI network. **(A)** Venn diagram of cross-talk genes from schizophrenia-related genes and two groups of DEGs, and 82 cross-talk genes were screened out. R vs C: RPD vs Control. K vs C: Knockdown vs Control. SCZ: schizophrenia. **(B)** GO analysis of the cross-talk genes. The bubble plot showed the top 10 processes in BP, and the only 2 processes in MF. There was no process enriched in CC. **(C)** KEGG enrichment analysis of the cross-talk genes. The bubble plot showed only the top four pathways were significantly enriched. **(D)** The STRING database was used to construct the PPI network of 82 cross-talk genes and the Cytoscape software was used to visualize the PPI network and screen the hub genes. The degree score was calculated for every gene, and the larger the circle and the darker the color meant the higher the degree score. p.adj < 0.05 was considered significant.

A total of 14,239 genes related to schizophrenia were collected from GeneCards, OMIM, and DisGeNET, deduplicated and combined to form a target gene dataset. Then, the SCZ-related genes were intersected with the above two sets of DEGs, and 82 cross-talk genes were screened out (Figure 4A). Subsequently, GO and KEGG analyses were performed. The GO analysis revealed that these 82 cross-talk genes were mainly enriched in bone mineralization, biomineral tissue development, and biomineralization (Figure 4B). According to KEGG pathway enrichment analysis, only 4 pathways were significantly enriched including Rap1 signaling pathway, Regulation of actin cytoskeleton, Calcium signaling pathway, and JAK-STAT signaling pathway (Figure 4C). To analyze the interaction among the proteins corresponding to the 82 genes, the STRING database was used to construct a PPI network. The software Cytoscape was then used to visualize the PPI network. The PPI network consisted of 43 nodes and 114 edges, 31 nodes and 96 edges were remained after hiding the unconnected genes. The genes with higher degree scores were labeled in darker colors and larger circles (Figure 4D). The hub genes were screened by the degree score, of which the top 10 genes included CSF2, FGFR2, FGF1, FGFR3, HBEGF, CDH5, MMP3, ANGPTL4, F2R, and PTPN6. After reviewing relevant literature, we selected fibroblast growth factors (FGFs)-related genes (including FGFR2, FGF1 and FGFR3) as the key genes that might be the potential targets for TNIK affecting the effect of RPD.

## 4 Discussion

In this study, we identified a total of 2,126 DEGs in RPD group and 655 DEGs in *TNIK* knockdown group after transcriptomic analysis. After intersecting the SCZ-associated genes group with the above two DEGs groups, we screened out a total of 82 genes. For the first time, we investigated the molecular mechanisms of gene expression and pathways in human glioma cells following the effects of RPD. Through KEGG enrichment analysis, we found a significant enrichment of the pathway for galactose metabolism and parathyroid hormone synthesis, secretion, and action. On the other hand, *TNIK*, a gene of interest, was first explored to identify the potential mechanisms following its knockdown, and the significant enrichments of the collagen-containing extracellular matrix, signaling receptor activator activity, and PI3K-Akt signaling and Ras signaling pathways were found. More interesting, the study showed that both RPD treatment and *TNIK* knockdown had a significant impact on bone mineralization and calcium signaling pathway. Furthermore, the PPI network analysis indicated that *TNIK* might affect the adverse effect of RPD through FGF-related genes, such as FGFR2, FGF1, and FGFR3.

Risperidone is one of the widely prescribed antipsychotics; however, long-term use is usually accompanied by various side effects, such as weight gain, extrapyramidal symptoms, prolactin elevation, and osteoporosis. The complex mechanism of RPD is not yet fully understood and requires further research. Therefore, we have studied at the cellular level which pathways can be altered after RPD treatment through transcriptomic analysis. As result showed, KEGG pathway analysis only emphasized two pathways: galactose metabolism and parathyroid hormone synthesis, secretion, and action pathway. Regarding galactose metabolism, Yu et al. suggested an association between upregulation of galactose metabolism and postmenopausal osteoporosis by metabolome analysis (Yu et al., 2019). In addition, galactose was used to induce aging and osteoporosis in the rat model, which exhibited marked bone loss (Yu et al., 2019; Li et al., 2020). Regarding parathyroid hormone synthesis, secretion, and action pathway, 20 genes enriched in this pathway, and 18 genes were significantly upregulated, indicating that this pathway might be highly activated during RPD application. Parathyroid hormone (PTH) is a peptide hormone made up of 84 amino acids that is synthesized and secreted by the parathyroid glands. It is essential for the metabolism of calcium and phosphorus through direct effects on bone and kidney and indirect effects on the human intestine (Chen T. et al., 2021). PTH has been shown to result in catabolic effects on the skeleton via continuous stimulation, while intermittent stimulation can lead to osteoanabolic effects (Silva and Bilezikian, 2015). Therefore, PTH has the opposite effects on bone resorption and bone formation in different situations. Overall, our previous description pointed out that long-term use of RPD might promote the risk of osteoporosis due to elevated prolactin levels (Graham et al., 2011; Bishop et al., 2012). However, the present study provided a new perspective that RPD might result in osteoporosis by affecting galactose metabolism and parathyroid hormone synthesis, secretion, and action pathway.

In recent studies, TNIK has been implicated in multiple cellular signaling processes. Among them, the most reported is that TNIK is the downstream signal protein and nuclear coactivator of the Wnt/β-catenin signaling pathway to fully activate Wnt signaling and promote cell growth and invasion (Ma et al., 2022). Larhammar et al. demonstrated that TNIK regulates stress-induced c-Jun-N-terminal kinase (JNK) signaling in neurons, which affects nervous system development, axon regeneration, and neuronal degeneration (Larhammar et al., 2017). In addition, research has also proven a critical role for TNIK in canonical NF-kB signaling (Shkoda et al., 2012). In the present study, GO enrichment showed that DEGs produced by *TNIK* knockdown were mainly enriched in cellular components about collagen-containing extracellular matrix, cell-substrate adhesion, receptor-ligand activity, and signaling receptor activator activity. *TNIK* has been implicated in postsynaptic signaling and regulation of cell proliferation (Coba et al., 2012). Therefore, GO analysis results suggested that downregulated TNIK might affect the interaction between extracellular proteins secreted into the extracellular matrix and receptors on the cell membrane, thereby regulating the binding of receptors and ligands, as well as the signal transduction for cell-to-cell communication. KEGG enrichment analysis highlighted PI3K-Akt signaling pathway. Research has found that the PI3K-Akt signaling pathway is mediated by several targets for SCZ and antipsychotics, such as dopamine, serotonin, glutamate, neuregulin 1, dysbindin, and DISC-1 (Liu et al., 2016). For example, the dysfunction of dopamine D1 and/or D5 receptor signaling in SCZ is linked to the activation of PI3K/AKT signaling, which subsequently leads to the inactivation of GSK3 (Matsuda et al., 2019). In addition, study of animal models has suggested that the miR-25-3p-mediated TWIST1/PI3K/Akt/GSK3β signaling pathway is altered in the brain of schizophrenia model rat, and this alteration can be restored by RPD, indicating that this pathway might be a potential biomarker for SCZ and a possible target for antipsychotic treatment (Pan et al., 2023). Furthermore, TNIK has been shown to play a role in the PI3K-Akt signaling pathway by regulating Akt activation in gastric cancer growth (Yu et al., 2014). These results suggested that downregulated TNIK might affect the efficacy of RPD through the PI3K-Akt signaling pathway.

Finally, to further identify the proteins and/or pathways that downregulated TNIK and RPD treatment co-influence in SCZ, we made the intersection among the SCZ-associated genes and the above two sets of DEGs, and a total of 82 cross-talk genes were obtained. According to GO analysis on these 82 genes, it was found that these genes were primarily associated with bone mineralization, biomineral tissue development, and biomineralization, indicating that RPD medication and *TNIK* knockdown might have a combined effect on biomineralization processes in schizophrenia. Biomineralization is a natural process through which inorganic calcium (Ca) and phosphate (P_i_) precipitate to form biological hard tissues such as bones, teeth, and other hard tissues (Bhadada and Rao, 2021). In bone mineralization, calcium phosphate crystals are deposited onto collagen fibrils, providing the bone with hardness and flexibility, respectively (Hasegawa et al., 2022). The accretion of adequate mineral content is essential for normal bone mineralization (Couce and Saenz de Pipaon, 2021). The pathological deficiency in mineralization results in osteoporosis due to the reduction in bone density (Vimalraj, 2020). Moreover, the chronic use of antipsychotics, particularly for RPD, has been associated with impaired bone mineralization, partially mediated by hyperprolactinemia (Calarge et al., 2018). Therefore we hypothesized that RPD may cause abnormal bone mineralization by elevating the level of prolactin, eventually leading to osteoporosis. Regarding *TNIK*, it is known to be an essential activator of the Wnt signaling pathway. The Wnt signaling pathway has been reported to promote bone formation by enhancing osteoblastic proliferation and differentiation, whereas aberrant activation of this pathway leads to altered osteocyte mineralization (Zhou et al., 2019; Palmer et al., 2021). Thus, the effect of downregulated TNIK on bone mineralization may be achieved by influencing the activation of the Wnt signaling pathway. Through KEGG pathway analysis, calcium signaling pathway was one of the significantly enriched pathways. Calcium signaling pathway is a crucial component of mechanisms that regulate neuronal excitability, information processing, and cognition, and dysregulation of calcium signaling has been implicated in the development of schizophrenia. Study suggested that the DISC1 gene may contribute to schizophrenia by interacting with calcium signaling pathways in the brain (Zhang et al., 2023), and variations in calcium channel signaling genes were also identified to play a role in the pathophysiology of schizophrenia through an extensive GWAS analysis (Cross-Disorder Group of the Psychiatric Genomics Consortium, 2013). Additionally, the extracellular level of ionic calcium may be one of the critical determinants for bone mineralization based on the chemical structure of bone minerals (Murshed, 2018). These results collectively suggested that both RPD and downregulated TNIK may affect the biological process of bone mineralization.

After analyzing the PPI network of 82 cross-talk genes and reviewing relevant literature, we screened three FGF-related genes (FGFR2, FGF1, and FGFR3) from the top 10 hub genes, and these three genes might be the potential targets through which *TNIK* might impact the adverse effect of RPD. In human, FGF family consists of 22 FGFs and 5 fibroblast growth factor receptors (FGFRs). The FGFs and their receptors have been reported to play an essential role in neurodevelopment and psychopathology, including SCZ (Terwisscha van Scheltinga et al., 2013; Talaei et al., 2020). FGF1, widely expressed in the human central nervous system, is located in the regions that have been replicated for linkage with SCZ (Terwisscha van Scheltinga et al., 2010). FGFR2, which is involved in presynaptic organization during early development, as well as neuroprotection and repair during adulthood, has been identified as a potential susceptibility gene for SCZ (O’Donovan et al., 2009). Moreover, a postmortem brain study for patients with SCZ revealed a decrease in the expression of FGFR2 in the hippocampus and cingulate cortex (Terwisscha van Scheltinga et al., 2010). Interestingly, the result of this study showed that the expression of FGFR2 was significantly upregulated with RPD treatment. This indicated that FGFR2 might also be a target for RPD to exert its effect during SCZ treatment. Regarding FGFR3, there is little evidence to discuss the association between FGFR3 and SCZ. According to previous studies, perhaps of most plausible relevance to SCZ is that FGFR3 is expressed in glial cells of the diencephalon and myelencephalon during development, with expression patterns widening during adulthood (Terwisscha van Scheltinga et al., 2010). Furthermore, studies have substantiated the strong interaction between FGFs and the dopamine system, which is strongly implicated in SCZ pathophysiology and is the main target for RPD medication (Grace, 2016; Talaei et al., 2020). For example, in a model of Parkinson’s disease, it has been proven that FGFs can protect against the loss of dopaminergic neurons and promote neurotrophic activity on dopamine neurons both *in vitro* and *in vivo* (Liu et al., 2021). Specifically, numerous studies have found that FGF2 is involved in the development and maintenance of the brain’s dopamine system (Even-Chen et al., 2017; Grinchii et al., 2023). On the other hand, we found that FGF, in addition to PTH, is widely expressed in the human bone matrix and plays a role in regulating the formation and development of osteoporosis (Gao et al., 2023). Early studies in animal models found that system administration of FGF1 can prevent bone loss and promote new bone formation in ovariectomized rats (Dunstan et al., 1999). FGFR2, as a cell membrane surface receptor, plays a critical role in bone development and homeostasis. Via stabilizing FGFR2, Otubain1 can positively regulate osteogenic differentiation and mineralization in bone homeostasis (Zhu et al., 2023). Overexpression of this gene in bone marrow mesenchymal stem cells has also been found to enhance osteogenesis and promote regeneration of critical cranial bone defects (Zhou et al., 2023). FGFR3 is a negative regulator of endochondral bone development. In an ovariectomized mouse model, activated FGFR3 can suppress bone regeneration and bone mineralization (Kawashima et al., 2023). Through targeting FGFR3, MiR-99b-5p can exhibit inhibitory effects on osteoblast proliferation and differentiation, thereby affecting bone formation in osteoporosis progression (Ding et al., 2021). As with FGFR2, FGFR3 was also significantly upregulated after RPD treatment. Therefore, RPD medication may also increase the incidence of osteoporosis by affecting the expression levels of FGF-related genes. In summary, the above three genes might be potential targets through which *TNIK* knockdown might affect the molecular expression of RPD.

However, there are several limitations in this study. Firstly, we conducted this study based on the association studies of *TNIK* affecting the antipsychotic effect of RPD. Therefore, the neuronal cell lines could be more suitable than the U251 cell line for this study to disentangle the mechanism involved in the antipsychotic effect of RPD. Secondly, the genetic variants of *TNIK* have been reported to be associated with the treatment response of several antipsychotic drugs, but only RPD was involved in this study. In addition, the use of IC50 as the dose for RPD treatment lacks sufficient empirical support, and determining the optimal concentration of RPD treatment will require further exploration in U251 cells. Last, in this study, our initial goal was to investigate the pathways or targets through which TNIK downregulation might influence the efficacy of RPD. Ultimately, we identified potential targets indicating that TNIK downregulation affects the adverse effect of RPD on osteoporosis. Overall, to determine the exact mechanism of *TNIK* affecting the efficacy of antipsychotics, further researches are required to explore the biological functions of the potential targets using more scientifically rigorous experimental protocols.

## Data Availability

The raw data supporting the conclusions of this article will be made available by the authors, without undue reservation.
