# Supplementary figures and images for: Transcriptome analysis to explore the mechanism of downregulated TNIK influencing the effect of risperidone
Source: Front Pharmacol. 2024 Aug 23;15:1431923. doi: 10.3389/fphar.2024.1431923 (PMC11391209; doi:10.3389/fphar.2024.1431923)

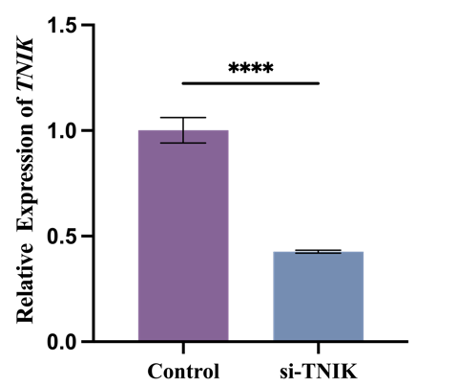

Supplement: Supplementary file 1 [file Image1.PNG]
